# Comparison of mental health outcomes of augmenting medications for patients with posttraumatic stress disorder: A national veterans affairs study

**DOI:** 10.1111/jep.13726

**Published:** 2022-06-16

**Authors:** Rachel Ranney, Shira Maguen, Anne Woods, Karen H. Seal, Thomas C. Neylan, Nancy Bernardy, Ilse Wiechers, Annie Ryder, Beth E. Cohen

**Affiliations:** 1 Veterans Affairs San Francisco Health Care System, San Francisco, California, USA; 2 Department of Psychiatry and Behavioral Sciences, San Francisco School of Medicine, University of California, San Francisco, California, USA; 3 Sierra Pacific Mental Illness Research Education, and Clinical Center, San Francisco, California, USA; 4 Northern California Institute for Research and Education, San Francisco, California, USA; 5 Department of Medicine, San Francisco School of Medicine, University of California, San Francisco, California, USA; 6 Veterans Affairs White River Junction Health Care System, White River Junction, Vermont, USA; 7 Department of Veterans Affairs, Northeast Program Evaluation Center, Office of Mental Health and Suicide Prevention, West Haven, Connecticut, USA; 8 Department of Psychiatry, Yale University School of Medicine, New Haven, New Haven, USA

**Keywords:** augmentation, medication, psychiatric hospitalization, PTSD, SRI, veteran

## Abstract

**Rationale:**

Posttraumatic stress disorder (PTSD) is highly prevalent among veterans. Many veterans with PTSD respond well to serotonin reuptake inhibitors (SRIs). Nonresponders may be prescribed augmenting medications, which are not as well-studied in PTSD.

**Aims and Objectives:**

We used Veterans Health Administration electronic records to compare mental health outcomes (PTSD symptoms and rates of mental health hospitalizations and psychiatric emergency room visits) in patients with PTSD who were prescribed four different groups of augmenting medications (atypical antipsychotics, mirtazapine, prazosin or tricyclic antidepressants) in addition to SRIs—from the year before to the year after the start of the augmenting medication.

**Method:**

We included data from 169,982 patients with a diagnosis of PTSD (excluding patients with comorbid bipolar or psychotic disorders) seen in Veterans Affairs care from 2007 to 2015 who were taking an SRI and filled a new prescription for one of the four augmenting medications for at least 60 days.

**Results:**

Patients evidenced minimal (<2%) reduction in PTSD symptoms and a larger reduction in psychiatric hospitalizations and psychiatric emergency room visits after receiving augmenting medications; this effect was largely similar across the four medication groups. Initiating augmenting medications was preceded by increases in PTSD symptoms, psychiatric hospitalizations and psychiatric emergency room visits. After initiating an augmenting medication, PTSD symptoms/hospitalizations/emergency room visits returned to baseline levels (before the start of the augmenting medication), but generally did not improve beyond baseline

**Conclusion:**

Importantly, these effects could be explained by regression to the mean, additional interventions or confounding. These findings should be further explored with placebo controlled randomized clinical trials.

## 1 | INTRODUCTION

Posttraumatic stress disorder (PTSD) causes substantial damage to the health of veterans and the general population. The lifetime prevalence of PTSD is approximately 6%–7% in the general population and 17%–22% in combat veteran populations in the United States.^1–3^ In addition to psychological distress and impairment, PTSD has been linked to higher rates of numerous health problems that can further impair patients function and quality of life.^4^ Healthcare costs are also substantial as patients with PTSD are more frequent users of the healthcare system compared to those without PTSD.^5,6^

Many studies have investigated the efficacy of medications for PTSD that are recommended by Veterans Affairs/Department of Defense practice guidelines.^7^ Serotonin reuptake inhibitors, including selective inhibitors such as sertraline, and serotonin/norepinephrine reuptake inhibitors such as venlafaxine (collectively referred to as serotonin reuptake inhibitors [SRIs] in this study), have been shown to reduce PTSD symptoms in randomized controlled trials.^8,9^ Unfortunately, many patients with PTSD will not respond to SRIs. In clinical trials, only 60% of patients on SRIs demonstrated significant improvements in PTSD symptoms, and only 20%–30% achieved remission.^10^

The Veterans Administration/Department of Defense Clinical Practice Guideline for the Management of Posttraumatic Stress (2017) currently does not recommend any medication for augmenting SRI pharmacotherapy for the treatment of PTSD due to a lack of and/or mixed evidence for augmenting medication; this represents a change from their previous recommended guidelines in 2010 which included guidance for medication augmentation in PTSD treatment.^7^ Despite these guidelines, recent research shows that many veterans with PTSD are prescribed augmenting medications.^11^ Treating patients with PTSD symptoms who have not responded to SRI pharmacotherapy can be especially challenging for prescribers in the absence of additional recommendations.^12^ The randomized controlled trial (RCTs) that have been conducted typically have stringent inclusion/exclusion criteria and may not represent the real‐world populations of treatment‐seeking patients. This is particularly important in PTSD, which is highly comorbid with other psychiatric and medical conditions. Prior trials have typically excluded older adults,^13^ underrepresented women^14^ and excluded patients with active suicidal ideation and substance dependence, common comorbidities among veterans with PTSD that may impact the efficacy of medications. Another limitation of current RCTs is the lack of comparisons between multiple augmenting medications. The lack of generalizable, real-world clinical data leaves patients and providers with little evidence to guide them when SRIs are insufficient. Thus, providers may prescribe additional medication when research evidence is absent, mixed or even suggests harm.^15^

Given this critical knowledge gap in the literature, we designed an observational study using national VA electronic health records to compare the impact of augmenting medications on PTSD symptoms, psychiatric hospitalizations and psychiatric emergency room visits. Though observational designs have limitations for making conclusions about causality, in the absence of clinical trials, they can detect signals of associated benefits or harms and can allow the study of a larger and more representative population. We selected augmenting medications that showed some evidence of benefit in clinical trials and/or had higher rates of use in VA populations, including prazosin, mirtazapine, specific atypical antipsychotics and tricyclic antidepressants.^16–23^ We compared mental health outcomes in the year before versus after starting the augmenting medication and also examined differences in effects by age and sex.

## 2 | METHODS

### 2.1 | Participants

This study was approved by the University of California, San Francisco Human Research Protection Program and the San Francisco Veterans Affairs Healthcare System Research and Development Committee. As the study involved secondary analysis of healthcare records, no participant contact was involved and informed consent was not required. To form this retrospective cohort, we selected patients who had received a diagnosis of PTSD (International Classification of Diseases, 9th Revision [ICD-9] Clinical Modification code 309.81 or ICD 10 F43.10,.11 or.12) in two or more outpatient encounters or one inpatient encounter between 1 January 2007 and 31 December 2015. Patients were selected from the Veterans Health Administration Corporate Data Warehouse (CDW) which is a comprehensive national repository of clinical and administrative data from within the VA and other external sources. We allowed at least 1 year of follow-up time after the augmenting medication was added for outcome assessment. We limited our study to veterans who were prescribed SRI medication for PTSD, including sertraline, paroxetine, venlafaxine, fluoxetine, fluvoxamine, citalopram, escitalopram, desvenlafaxine, and duloxetine and had one medication from the following four medication groups added: atypical antipsychotic (quetiapine, risperidone or olanzapine), prazosin, mirtazapine, and tricyclic antidepressants (amitriptyline or imipramine). We considered the initial date the augmenting medication was filled as the ‘index date’ and evaluated the change in mental health from 1 year before 1 year following the index date (see Supporting Information: Figure 1).

Patients were included if they had filled at least 30 days of SRI in the year before starting an augmenting medication. Patients were required to be free of the augmenting medication for 180 days leading up to the initiation date of the augmenting medication to capture new medications. The new augmenting medication had to be filled for at least 60 days within a 120-day period and the patient needed to receive a prescription of an SRI for at least 60 days in the year after initiating the augmenting medication. Though these were our minimum requirements, we found that most participants received both the SRI and augmenting medication for substantially longer. The median and interquartile range of days of overlap of both the SRI and augmenting medication fills were: atypical antipsychotics (171; 90–270 days), mirtazapine (134; 70–251), prazosin (176; 90–272) and tricyclics (132; 78–240). The median and interquartile range for total days on the augmenting medications during the post-index year, without regard to concurrent SRI use, were: atypical antipsychotics (210; 120–311 days), mirtazapine (180; 90–307), prazosin (210; 120–310) and tricyclics (180; 90–291).

**FIGURE 1:**
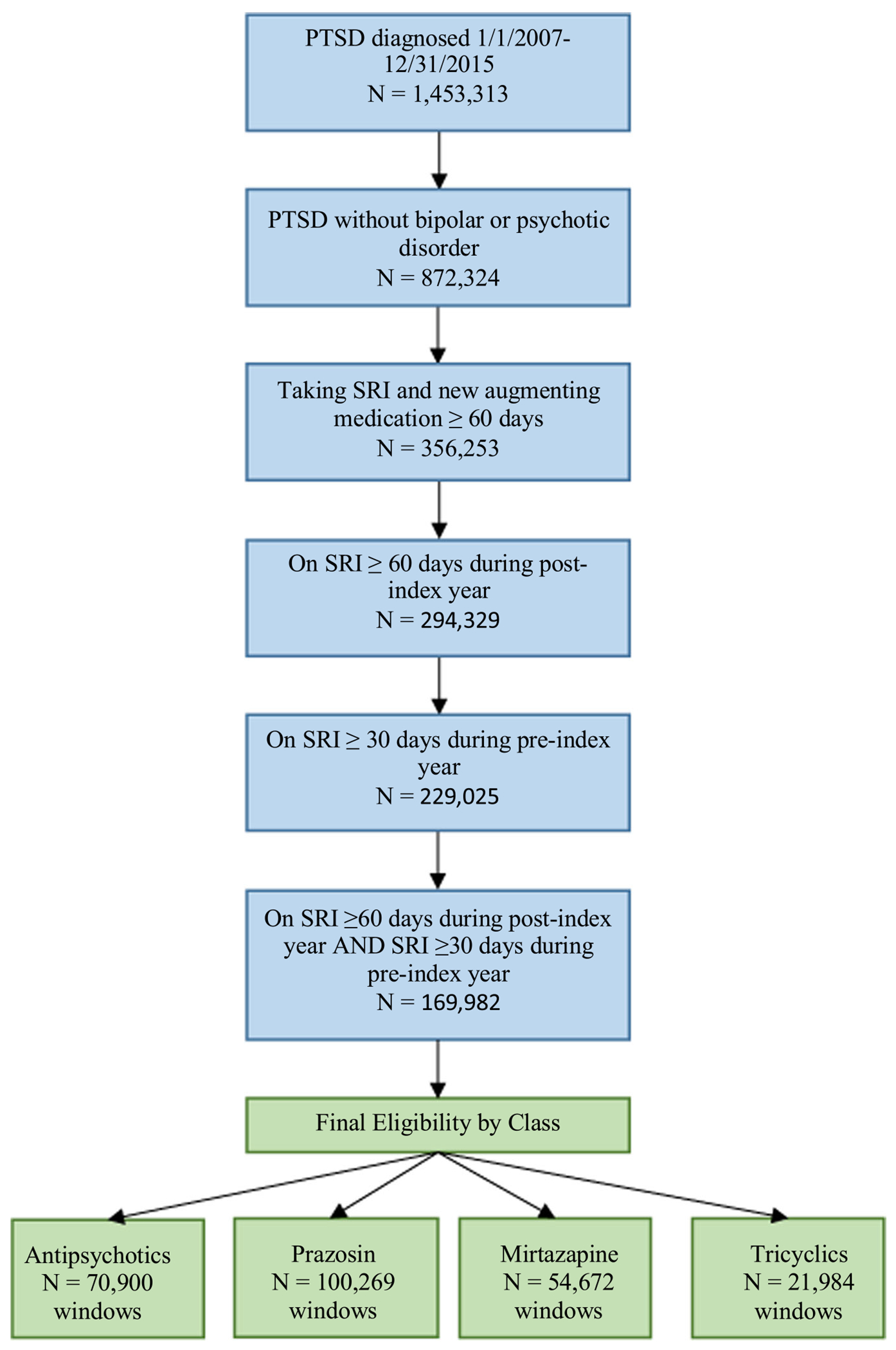
Cohort construction

Because polypharmacy is common in patients with PTSD,^24^ we allowed patients to contribute data to more than one medication class and also to fill other classes of augmenting medications as long as only one was ‘new’ based on our definition. Veterans with a diagnosis of bipolar affective disorder or psychotic disorders (using two or more outpatient encounters or one inpatient encounter) were excluded as these conditions could confound the data on the use of antipsychotic medications for the indication of PTSD. After all exclusions, 169,982 veterans remained in our final sample (see Figure 1).

### 2.2 | Sources of data

We used data from the Veterans Health Administration CDW. The CDW contains information on VA inpatient and outpatient visits and associated clinical diagnoses, information on non-VA visits reimbursed by VA, vital signs, VA pharmacy records, laboratory data and other patient-level variables. The Pharmacy Benefits Management database contained additional detailed information about medication fills that we used to determine eligibility. We used VA pharmacy prescription records that included medication type, dose, date of fill, amount dispensed and days supply to create a record of medications that patients possessed on a given calendar day. We used previously described methods^25^ to account for availability of medications, creating an array of days during the index period with 1 indicating the medication was available and 0 indicating it was not available. To account for overlapping days in supply (which happen when a patient requests refills of prescriptions before their current supply has run out), we credited forward to the day with the next 0.

### 2.3 | Measures

#### 2.3.1 | PTSD checklist (PCL)

The PCL assesses prior-month PTSD symptoms and is routinely administered in mental health treatment settings within the VA to track patient progress during the course of mental health treatment, with automated templates to facilitate data collection.^26^ In the current study, we analysed available data from the PCL assessing PTSD symptoms included in the Diagnostic and Statistical Manual of Mental Disorders, Fourth Edition. Only 3% of PCL assessments available used the updated PCL-5 (which assesses PTSD symptoms included in the DSM-5), and these were excluded given the differing questions and scoring ranges of the two versions. The PCL is the most commonly used self-report measure of PTSD symptoms with over 20 validation studies confirming its diagnostic accuracy.^27,28^ It correlates strongly with other measures of PTSD symptoms and demonstrates high diagnostic efficiency compared to gold standard diagnostic clinical interviews.^27,28^

#### 2.3.2 | Service utilization indicators

Psychiatric hospitalizations and emergency room visits were identified using ICD-9 and 10 codes from primary discharge diagnoses from hospitalizations and primary diagnoses from emergency room visits using a coding algorithm provided by the VA Northeast Program Evaluation Centre.^29^ Psychiatric hospitalizations and emergency room visits were measured in events per 100 person years. In the pre-index year, 8% of patients had a psychiatric hospitalization and 6% of patients had a psychiatric emergency room visit. In the post-index year, 5% of patients had a psychiatric hospitalization and 4% of patients had a psychiatric emergency room visit (see Supporting Information: Table 1 for changes in percent of patients hospitalized by augmenting medication group).

#### 2.3.3 | Covariates

We identified multiple covariates that were important to consider in analyses of medication use and mental and physical health outcomes based on prior research.^30,31^ Covariates included sociodemographic information, service utilization factors, mental health conditions and medical comorbidities. Sociodemographic information collected for this study was age, sex, race/ethnicity, marital status, rural versus urban status. Mental health and medical comorbidities collected for the study included depression, personality disorders, anxiety disorders, insomnia, substance abuse/dependence, alcohol abuse/dependence, traumatic brain injury, obesity, dyslipidemia, diabetes, hypertension, ischemic heart disease, congestive heart failure and cerebrovascular disease determined by ICD-9/10 code diagnoses (see Supporting Information: Table 2 for coding algorithms). The Charlson Comorbidity Index Score was also calculated as a general measure of health status for patients. Service utilization factors collected included distance to nearest VA medical centre, type of centre (community-based outpatient clinic vs. medical centre), primary care utilization (number of primary care visits), level of VA service connection (a rating from 0% to 100% that reflects medical conditions related to military service and impacts patient costs for VA care) and mental health utilization (number of mental health visits).

### 2.4 | Statistical analyses

Analyses were performed with SAS Enterprise Guide version 7.15 (SAS Institute) with a two-tailed α of 0.001 (adjusting the standard *p*-value to account for multiple hypotheses tested). We analysed changes in PCL using generalized linear models. We used antipsychotic medications as the reference group as they were hypothesized to have the smallest benefit based on existing clinical trials. We tested the interaction effect of time (from pre- to post-index year) and augmenting medication group on PCL scores, while adjusting for covariates listed above.

We analysed changes in psychiatric hospitalizations and emergency room visits using generalized linear mixed models with a within-subject random effect to account for multiple windows within each patient (see below). A Poisson distribution was used as these visits were count data. We tested the interaction of time (pre- to post-index) and augmenting medication group on the number of the psychiatric hospitalizations and emergency room visits.

We used multiple methods to adjust for confounding in our models. For traditional methods, covariates described above were added to the models. We also conducted propensity score weighted analyses to address imbalances in covariates in the four medication groups. For propensity score weighting, we estimated propensity scores with all covariates to predict probability of assignment to the four augmenting medication groups using generalized boosted regression with R Package ‘twang’. We assessed the propensity-weighted balance of covariates among the four groups using weighted *X*^2^ and *t*-tests. Propensity scores were normalized to sum to the total number of windows (the year-long units of observation time), then the normalized propensity score was used as an observation weight for each analytic window. We also evaluated overlap among the treatment groups using standard graphical and numerical diagnostics. We examined the distribution of weights and conducted sensitivity analyses trimming weights at the 95%.

We also completed several additional sensitivity analyses. We adjusted models for percentage of time on augmenting treatment during the post-index year to account for variations in time on medications. In another sensitivity analysis, we allowed patients to contribute data at only one time point and restricted to patients using only a single class of augmenting medication during the 2-year study observation period. For all primary analyses, we present the propensity weighted analyses.

### 2.5 | Missing data

Missing data were low for covariates. We expected the PCL would only be available on a subset of the patients given it is not routinely assessed at every psychiatric visit. PCL measures were available for 26.3% of the pre-index and 26.1% of the post-index windows. This was consistent with availability from prior VA EHR studies.^32^ For the PCL, the amount of missing data was too large to apply imputation. Despite these high rates of missing data, power analyses demonstrated that power was sufficient to detect effects well below thresholds of clinical significance.

## 3 | RESULTS

### 3.1 | Study cohort construction and patient characteristics

Before applying the propensity score weights, patients in the four augmenting medication groups were largely similar in terms of demographics, comorbidities, prescribing facility characteristics and service utilization. Due to the large size of the data set, even small variations are statistically significant (see Supporting Information: - Table 3). However, some notable clinically significant differences were the larger proportion of women in the group prescribed tricyclics (15.2%) versus in the other medication groups (7.7%–8.2%) and the increased likelihood of substance use disorders in those prescribed antipsychotics (36.6%) or mirtazapine (34.9%) versus those prescribed prazosin (32.4%) and tricyclics (26.2%). Propensity score weighting balanced these and other characteristics (Table 1). All analyses presented below are propensity score weighted; see Supporting Information: Table 4 for unweighted results.

### 3.2 | PTSD symptom score

**FIGURE 2:**
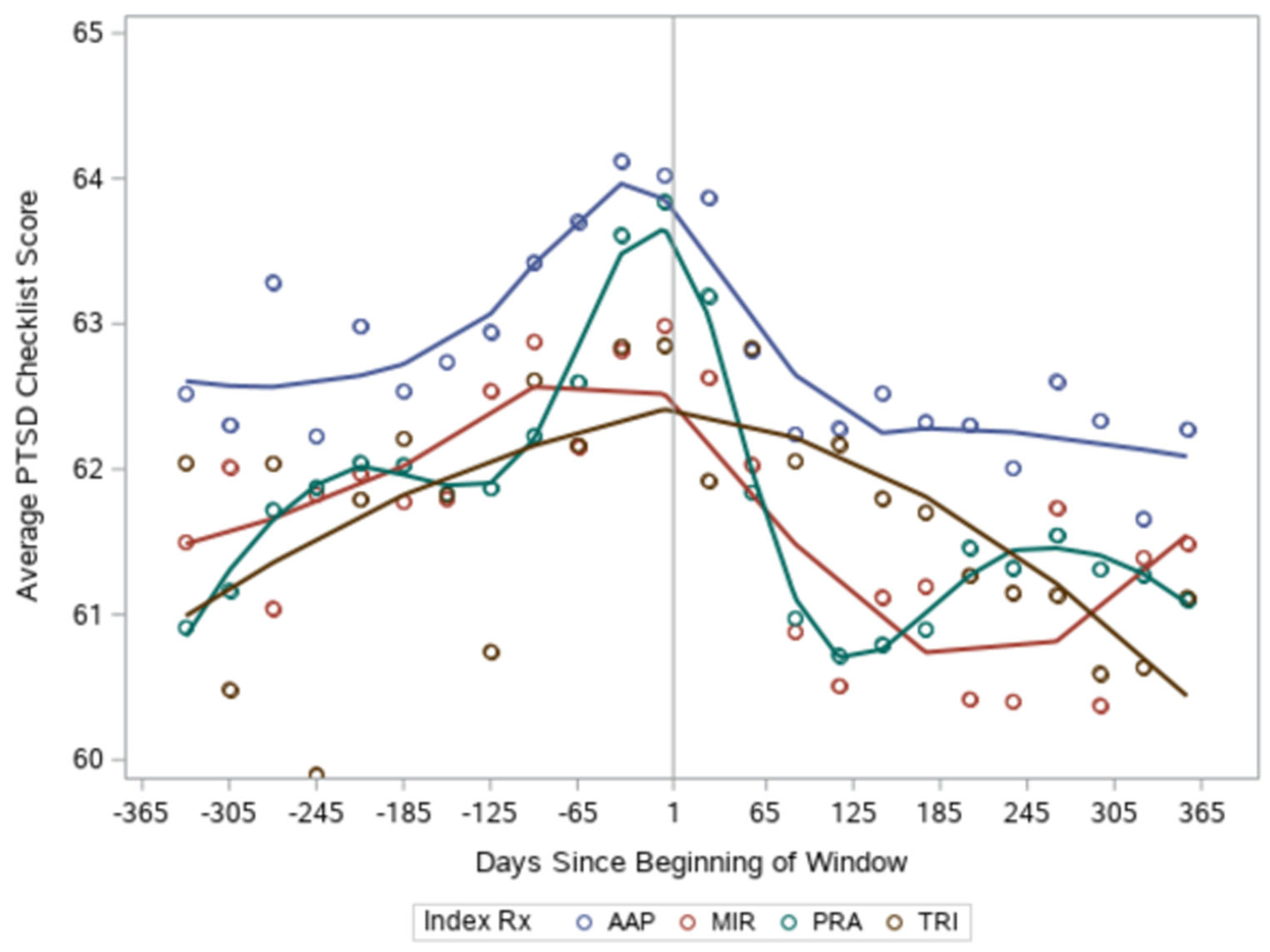
PCL score over the pre- and post-index year by augmenting medication group

Mean PCL scores in the pre-index year were in the low 60s, indicating moderate PTSD (Table 2). Across all four augmenting medication groups, PTSD symptom scores changed minimally from the pre- to post-index year, decreasing by approximately 1 point (Table 2), with no significant medication group differences (Table 3). Given the small change in PCL score when using the averages of the pre- and post-index year, we graphed all available timepoints of PCL over the 2-year period to further evaluate temporal changes. Figure 2 displays smoothed lines representing the population mean PCL score in each of the four groups over the 2-year study period. For each medication group, PTSD symptoms increase before the addition of the augmenting medication and then decrease to roughly the baseline level after 3–4 months. Beyond this point, symptoms do not continue to improve.

### 3.3 | Mental health emergency room visits and hospitalizations

**FIGURE 3:**
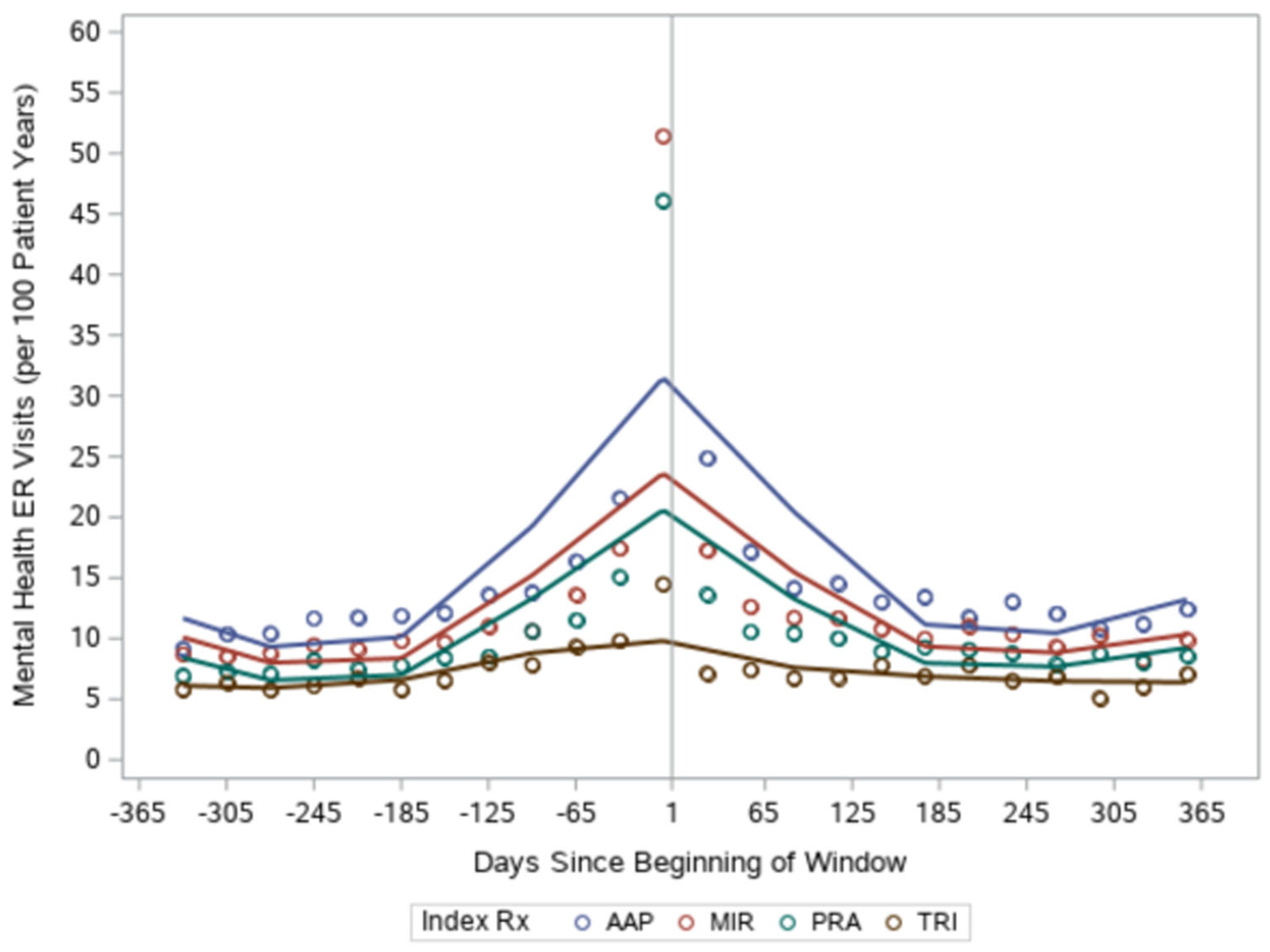
Mental health emergency room visits over the pre- and post-index year by augmenting medication group

**FIGURE 4:**
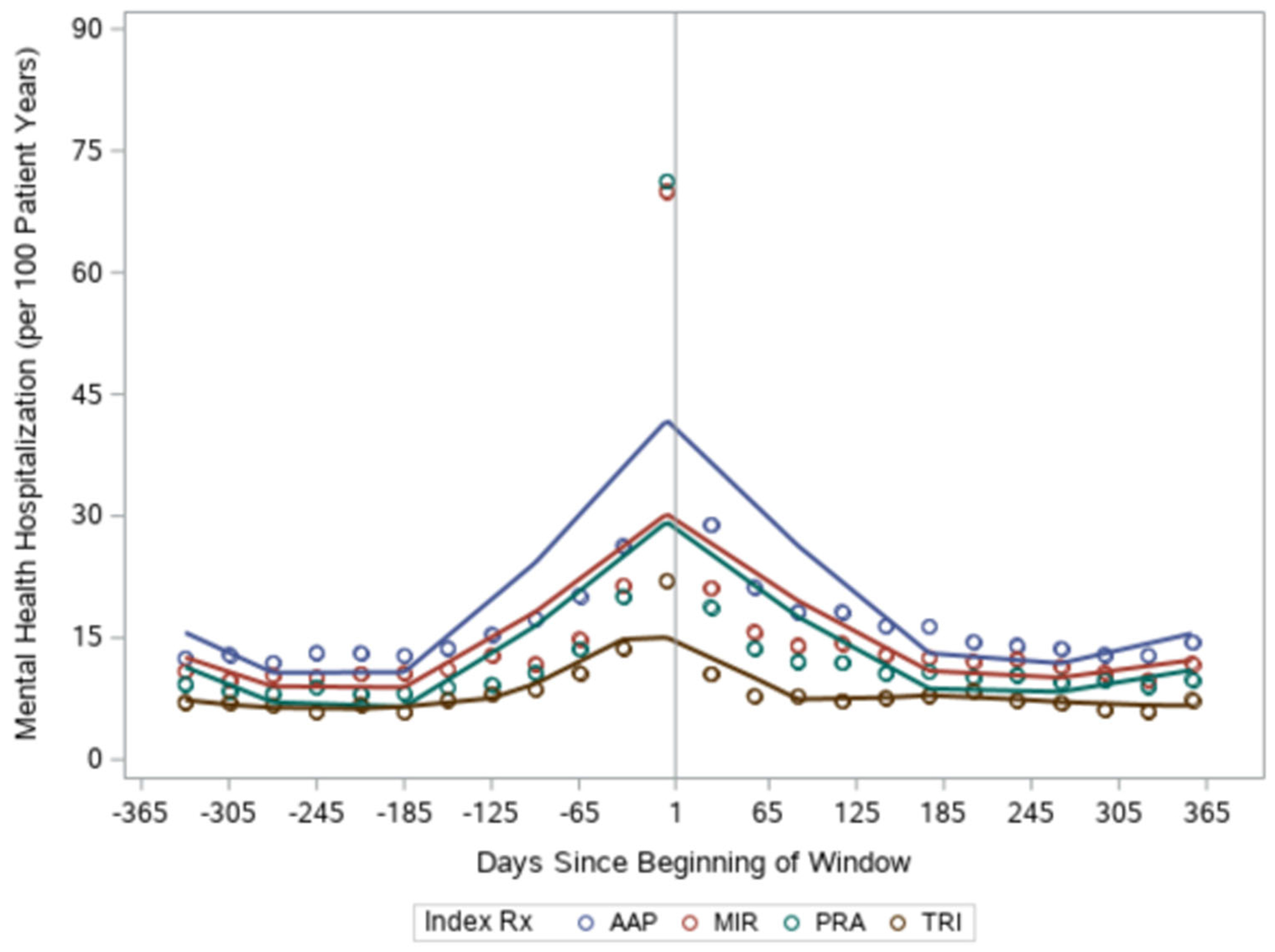
Mental health hospitalizations over the pre- and post-index year by augmenting medication group

We found the mental health emergency visits were highest among the antipsychotic group and lowest among those augmented with tricyclics (in both the pre-index year and post-index year, see Table 2). All groups had declines in rates of mental health emergency room visits (relative decreases of 16%–20%) in the year following addition of the augmenting medication. There were no significant differences by medication class in the decrease in emergency room visits (Table 3). Mental health hospitalizations followed a similar pattern, with the antipsychotic group demonstrating the most hospitalizations, the tricyclic group demonstrating the least hospitalizations and hospitalization dropping in all groups in the year following the addition of an augmenting medication (relative decreases of 21%–26%, see Table 2)—with no differences between medication groups (Table 3). These more dramatic reductions in mental health emergency visits/hospitalizations contrast with the minimal change in PTSD symptom score. As with PTSD symptoms, we conducted additional analyses to more closely examine the temporal pattern of emergency room visits and hospitalizations. We found that the augmenting medication started after a spike in mental health emergency visits/hospitalizations in all groups except tricyclics (Figures 3 and 4). After augmentation, the rate returned to baseline levels.

### 3.4 | Subgroup differences

We also aimed to examine subgroup differences, examining sex and age. This involved repeating all analyses for each of the subgroup categories. For each outcome tested, we examined two-way interactions (e.g., time × age interaction ran separately for each medication group). We also examined three-way interactions on each outcome tested (e.g., time × age × medication group).

We did not find any clinically significant subgroup differences in PCL scores, mental health hospitalizations or emergency room visits; please see our Supporting Information: Tables 5–7 for further detail.

### 3.5 | Sensitivity analyses

We conducted numerous sensitivity analyses to test the robustness of our conclusions and to further explore the impact of dose and duration of medication exposure. Using a traditional adjustment approach, which included the covariates in Table 1 rather than a propensity weighted model, did not change our findings (see Supporting Information: Table 4). Adjusting for time on augmenting medications, dosage, exposure to other classes of augmenting medications and repeating analyses in a restricted sample using only a single class of augmenting medication did not change our findings (see Supporting Information: Tables 8 and 9 for further detail).

## 4 | DISCUSSION

In this large observational study using data from nearly 170,000 VA patients with PTSD who were prescribed serotonin reuptake inhibitors, we found that patients had statistically significant but clinically small improvements (<2% decline) in PTSD symptoms after receiving augmenting medications and that the effect was similar across drug classes. We observed larger reductions in rates of emergency room visits and hospitalizations for mental health conditions (11%–25% decline). We found that medications were prescribed after increases in symptoms and emergency room visits/hospitalizations. This suggests that providers may be adding medications in response to symptom flares or problems with comorbid mental health conditions as a stabilization strategy, rather than using these for patients who did not respond to SRIs. Following the initiation of the augmenting medication, mental health outcomes tended to return to baseline levels without improving further in all four medication groups. Importantly, given the observational nature of our study and lack of an untreated ‘control’ group, it is not clear whether the return to baseline symptom level is a result of the augmenting medication or of regression to a baseline mean over time. Regression to the mean occurs when measurements are more extreme, with the next sampling of the same variable likely to be closer to the mean. Given that these augmenting medications seem to be prescribed in response to symptom flares, this return to baseline may also be accounted for by other interventions applied at the same time as the augmenting medication to reduce symptoms, such as psychotherapy and case management.

When we compared effects across subpopulations, we found that mental health outcomes were largely similar across age and gender. No previous studies have investigated gender differences in response to augmenting medications for PTSD, but one previous study found a lack of gender differences in response to primary SRI treatment for PTSD.^33^ Our results add important information given existing trials have tended to enroll homogenous populations, such as exclusively male veterans. Regarding age, no previous studies have investigated age differences in response to augmenting medications for PTSD. One study of nonveterans did not find a significant effect of age on treatment outcomes for primary SRI treatment for PTSD.^33^

It is challenging to compare our findings to prior studies as none have directly compared the mental health outcomes of these medications. However, there have been some RCTs that compare one of these augmenting medications to placebo. The largest RCTs investigating atypical antipsychotics as an augment to SRI medication for PTSD found no support for clinically significant improvements in PTSD symptoms compared to placebo.^16–19^ RCTs have not tested prazosin as an augmenting medication for PTSD; RCTs testing prazosin as a primary medication for PTSD have yielded conflicting findings regarding efficacy over placebo.^20–22,34–37^ Regarding mirtazapine, one study demonstrated that this medication may be efficacious as an augmenting medication for PTSD^23^; however, more studies are needed to confirm this finding.

Due to the lack of strong evidence that augmenting medication leads to any benefit, the Veterans Administration/Department of Defense Clinical Practice Guideline for the Management of Post-traumatic Stress (2017) no longer recommends any augmenting medication for PTSD; the previous guidelines in 2010 recommended mirtazapine, prazosin and tricyclic antidepressants as augmenting medications, but did not recommend atypical antipsychotics.^7^ A previous study of PTSD prescribing practices in the VA found the use of several augmenting classes examined in the current study, including atypical antipsychotics and tricyclics, has declined over time. However, many patients with PTSD continue to receive these medications.^11^

Regarding clinical implications, the more dramatic declines in hospitalizations and emergency room visits we found compared to the minimal change in PTSD symptoms may suggest that these medications could be useful in a select population to stabilize patients in crisis situations, but this would need to be confirmed in controlled trials. Also, it is unclear whether augmenting medications would be helpful for long-term use, and the metabolic and cardiovascular risks associated with longer term use of some types of augmenting medications raise important concerns.^30,38–40^ Additionally, polypharmacy is common in patients with PTSD,^24^ and this increases potential for risk. Given the similar mental health outcomes among these augmenting medications, differing side effect profiles of these medications are an important consideration. Further research, including placebo-controlled clinical trials in settings of acute symptom flares, is needed to determine whether some patients may benefit from augmenting medications. Trials could also compare augmenting medications to nonpharmacological interventions, such as psychosocial stabilization interventions and evidence-based psychotherapy for PTSD, which do not carry the health risks of medication.^41,42^ Evidence-based psychotherapy for PTSD has been found to help improve complex symptoms such as suicide risk and psychotic symptoms.^43–46^ Thus, clinicians may consider offering these treatments to patients as soon as possible to help prevent crisis situations as well as improve PTSD symptoms.

There are several limitations to our study. First, our study used observational data. Though we used traditional methods and propensity score weighting to help reduce confounding, patients were not randomized to the augmenting medications and we did not have a control group continuing SRI without addition of an augmenting medication. This limits our ability to draw conclusions about the causality of the associations we observed as they could be due to temporal changes, a placebo effect, regression to the mean or unmeasured confounding. Second, this cohort includes veterans who served post-9/11, were mostly male, using VA care and so may not be generalizable to other populations. Third, although ascertainment of medication use was thorough, we relied on prescription fill data to determine when patients possessed medications, but this does not represent actual use, and we did not have access to non-VA prescription data. The indication for prescriptions is not included in the data available, so it is possible that some of these medications were prescribed to target conditions other than PTSD, and we were not able to control for all of these indications as covariates in models. Fourth, while data were complete for covariates and psychiatric emergency room visits/hospitalizations, a substantial number of assessment windows did not have PTSD symptom scores in the required time frame before and following augmenting medication initiation. We retained substantial power to detect clinically meaningful changes in PTSD symptoms given the large sample size and missing data were similar in the four augmenting medication groups. Fifth, the current study relied on self-reported PTSD symptoms from the PCL; future studies should include clinical interviews.

Future research would benefit from RCTs comparing various augmenting medications for PTSD with a control condition of participants assigned to continue SRIs without an augmenting medication. These prospective designs, which might include point of care trials, may also include clinical interviews to comprehensively assess PTSD symptoms. Additionally, because these medications seem to have been prescribed in response to a flare in PTSD symptoms, trials may enroll participants who are experiencing a flare in PTSD symptoms to test efficacy of these medications in cases when they are most likely to be prescribed. Future retrospective studies may include stratification by clinical group (e.g., stratification by inpatient vs. outpatient). Investigating an inpatient participant group separately may better allow for comparisons between participants receiving versus not receiving an augmenting medication. Retrospective studies may also stratify by clinical symptoms, such as patients with nightmares, to elucidate potential moderating effects of medications for patients with different presentations.

There has been great controversy in the field about how to treat the large number of patients with PTSD who do not have an adequate response to SRIs. We found that the augmenting medications were prescribed after increases in PTSD symptoms and mental healthcare utilization in this national sample of veterans. Following the initiation of an augmenting medication, the rates of these outcomes returned to baseline levels in the population over the next few months. These augmenting medications may benefit patients through stabilization during crises, but we did not see evidence for long-term symptom benefits. Clinical trials are needed to fully understand whether these medications were helpful for stabilizing patients during a flare in psychiatric symptoms or whether our findings are better attributed to other factors. This is especially important to evaluate given the health risks of some augmenting medications. Well-designed clinical trials can help the many patients with inadequate treatment responses to SRIs make more informed decisions about their care.

## Supplementary Material

Supplement

Additional supporting information can be found online in the Supporting Information section at the end of this article.

## Figures and Tables

**TABLE 1:** Patient characteristics by augmenting medication class: Propensity score weighted

	Antipsychotics	Mirtazapine	Prazosin	Tricyclics	*p* value
Sociodemographics					
Age	52.2/13.4	52.2/15.2	52.2/11.2	52.2/23.6	0.99
Male	91.5	91.4	91.5	91.4	0.88
Female	8.5	8.6	8.5	8.6	
Race (White)	73.3	73.4	73.2	73.5	0.89
Race (Black)	19.2	19.2	19.2	19.1	
Race (other)	7.5	7.5	7.6	7.4	
Ethnicity (Hispanic)	7.3	7.3	7.4	7.1	0.44
Ethnicity (non-Hispanic)	90.6	90.7	90.6	90.9	
Ethnicity (unknown)	2.1	2.1	2.1	2.1	
Marital status (married)	57.9	57.9	58.1	58.1	0.96
Marital status (never married)	12.5	12.5	12.4	12.3	
Marital status (other)	29.6	29.6	29.6	29.6	

Comorbidities					
Major depressive disorder	36.8	36.8	36.7	36.9	0.95
Personality disorder	5.1	5.0	5.0	4.9	0.69
Generalized anxiety disorder	7.7	7.7	7.6	7.4	0.16
Insomnia	18.1	18.2	18.1	18.3	0.72
Substance abuse/dependence	33.7	33.7	33.5	33.3	0.41
Alcohol abuse/dependence	27.7	27.7	27.8	27.4	0.58
Traumatic brain injury	6.1	6.1	6.1	6.3	0.23
Obesity	24.8	23.0	29.0	26.6	<0.0001
Dyslipidemia	54.0	55.1	56.1	55.4	<0.0001
Diabetes	22.2	21.9	24.6	24.9	<0.0001
Hypertension	53.8	53.9	55.4	54.4	<0.0001
Ischemic heart disease	3.8	3.8	3.3	3.4	<0.0001
Congestive heart failure	3.4	3.5	3.0	2.9	<0.0001
Cerebrovascular disease	1.2	1.2	1.1	1.1	0.05
Charlson Comorbidity Index score	1.2/1.6	1.2/1.8	1.2/1.3	1.2/2.8	0.80

Service utilization factors					
Primary facility (VA medical centre)	61.8	61.7	61.8	61.7	0.95
Primary facility (community-based outpatient clinic)	38.2	38.3	38.2	38.3	
Primary care utilization (# visits in pre-index year)	3.8/3.5	3.8/3.9	3.8/2.9	3.8/6.0	0.77
Mental health utilization (# visits in pre-index year)	16.7/27.5	16.6/31.1	16.7/23.1	16.4/47.2	0.26
Drive time to nearest VA	23.3/22.4	12.3/23.9	23.4/21.1	23.2/36.8	0.40
VA service connection level	52.5/33.5	52.5/38.1	52.6/28.1	52.8/59.6	0.39

*Note*: Values are shown as percent for categorical variables and mean/standard deviation for continuous variables.

**TABLE 2:** Propensity score weighted changes in each outcome by augmenting medication group

Medication group	Pre-index year	Post-index year	Change (post minus pre)	95% CI for absolute change	% change	*p* value
PTSD checklist score						
Antipsychotics	63.04	62.25	−0.79	(−1.04, −0.54)	−1.26	<0.0001
Mirtazapine	62.07	61.30	−0.77	(−1.06, −0.49)	−1.25	<0.0001
Prazosin	62.71	61.62	−1.09	(−1.28, −0.91)	−1.74	<0.0001
Tricyclics	62.18	61.26	−0.93	(−1.41, −0.44)	−1.49	0.0002

Mental health ER visits (# per 100 person years)						
Antipsychotics	15.92	12.77	−3.15	(−3.80, −2.51)	−19.80	<0.0001
Mirtazapine	13.94	11.15	−2.80	(−3.46, −2.14)	−20.06	<0.0001
Prazosin	12.83	10.04	−2.78	(−3.28, −2.29)	−21.70	<0.0001
Tricyclics	9.71	8.13	−1.58	(−2.42, −0.75)	−16.29	0.0002

Mental health hospitalizations (# per 100 person years)						
Antipsychotics	19.96	15.17	−4.79	(−5.41, −4.18)	−24.01	<0.0001
Mirtazapine	16.83	13.25	−3.58	(−4.23, −2.93)	−21.26	<0.0001
Prazosin	16.30	12.00	−4.30	(−4.77, −3.82)	−26.37	<0.0001
Tricyclics	12.45	9.31	−3.14	(−3.99, −2.29)	−25.19	<0.0001

Abbreviations: ER, emergency room; PTSD, posttraumatic stress disorder.

**TABLE 3:** Propensity weighted models for changes in mental health outcomes from pre- to post-index year by augmenting medication

PTSD checklist score			Mental health ER visits (per 100 person years)			Mental health hospitalizations (per 100 person years)		
Diff. to ref	95% CI	*p* value	Diff. to ref	95% CI	*p* value	Diff. to ref	95% CI	*p* value
Mirtazapine								
0.003	(−0.36, 0.37)	0.99	0.35	(−7.04, 7.75)	0.93	1.22	(−0.68, 3.11)	0.21

Prazosin								
−0.32	(−0.67, 0.03)	0.07	0.37	(−0.72, 1.45)	0.50	0.49	(−0.39, 1.38)	0.27

Tricyclics								
−0.15	(−0.5, 23)	0.45	1.57	(−1.22, 4.36)	0.27	1.66	(−4.81, 8.12)	0.62

*Note*: Model shows coefficient for difference in change in outcome score from pre- to post-index year as compared to reference group of antipsychotics. Abbreviations: ER, emergency room; PTSD, posttraumatic stress disorder.

## Data Availability

VA data reside in the VA Corporate Data Warehouse and are not publicly available. VA investigators can apply to get access through the VA Informatics and Computing Infrastructure (VINCI).

## References

[1] Koenen KC, Ratanatharathorn A, Ng L, Posttraumatic stress disorder in the world mental health surveys. Psychol Med. 2017;47(13):2260–2274. doi:10.1017/S0033291717000708 28385165 PMC6034513

[2] Goldberg J, Magruder KM, Forsberg CW, Prevalence of post-traumatic stress disorder in aging Vietnam-era veterans: Veterans Administration Cooperative Study 569: course and consequences of post-traumatic stress disorder in Vietnam-era veteran twins. Am J Geriatr Psychiatry. 2016;24(3):181–191. doi:10.1016/j.jagp.2015.05.004 26560508 PMC5928006

[3] Goldstein RB, Smith SM, Chou SP, The epidemiology of DSM-5 posttraumatic stress disorder in the United States: results from the National Epidemiologic Survey on alcohol and related conditions-III. Soc Psychiatry Psychiatr Epidemiol. 2016;51(8):1137–1148. doi:10.1007/s00127-016-1208-5 27106853 PMC4980174

[4] Magruder KM, McLaughlin KA, Elmore Borbon D, L. Trauma is a public health issue. Eur J Psychotraumatol. 2017;8(1):1375338. doi:10.1080/20008198.2017.1375338 29435198 PMC5800738

[5] Cohen BE, Gima K, Bertenthal D, Kim S, Marmar CR, Seal KH. Mental health diagnoses and utilization of VA non-mental health medical services among returning Iraq and Afghanistan veterans. J Gen Intern Med. 2010;25(1):18–24. 10.1007/s11606-009-1117-3 PMC2811589 19787409

[6] Haskell SG, Mattocks K, Goulet JL, The burden of illness in the first year home: do male and female VA users differ in health conditions and healthcare utilization. Womens Health Issues. 2011;21(1):92–97. 21185994 10.1016/j.whi.2010.08.001 PMC3138124

[7] VA/DoD Clinical Practice Guideline for the Management of Posttraumatic Stress Disorder. https://www.healthquality.va.gov/guidelines/MH/ptsd/VADoDPTSDCPGFinal012418.pdf. Accessed 25 October 2021.

[8] Hoskins M, Pearce J, Bethell A, Pharmacotherapy for posttraumatic stress disorder: systematic review and meta-analysis. Br J Psychiatry. 2015;206(2):93–100. doi:10.1192/bjp.bp.114.148551 25644881

[9] Ipser JC, Stein DJ. Evidence-based pharmacotherapy of post-traumatic stress disorder (PTSD). Int J Neuropsychopharmacol. 2012;15(6):825–840. doi:10.1017/S1461145711001209 21798109

[10] Alexander W. Pharmacotherapy for post-traumatic stress disorder in combat veterans: focus on antidepressants and atypical antipsychotic agents. P T. 2012;37(1):32–38. 22346334 PMC3278188

[11] Holder N, Woods A, Neylan TC, Trends in medication prescribing in patients with PTSD from 2009 to 2018: a National Veterans Administration Study. J Clin Psychiatry. 2021;82(3):20m13522. doi:10.4088/JCP.20m13522. 34004087

[12] Bernardy NC, Lund BC, Alexander B, Friedman MJ. Prescribing trends in veterans with post-traumatic stress disorder. J Clin Psychiatry. 2012;73(3):297–303. doi:10.4088/JCP.11m07311 22490256

[13] Watts G. Why the exclusion of older people from clinical research must stop. BMJ. 2012;344:e3445. 22613873 10.1136/bmj.e3445

[14] Krystal JH, Rosenheck RA, Cramer JA, Adjunctive risperidone treatment for antidepressant-resistant symptoms of chronic military service-related PTSD: a randomized trial. JAMA. 2011;306(5):493–502. 21813427 10.1001/jama.2011.1080

[15] Finley EP, Mader M, Haro EK, Use of guideline-recommended treatments for PTSD among community-based providers in texas and vermont: implications for the veterans choice program. J Behav Health Serv Res. 2019;46(2):217–233. doi:10.1007/s11414-018-9613-z 29748747

[16] Krystal JH, Pietrzak RH, Rosenheck RA, Sleep disturbance in chronic military-related PTSD: clinical impact and response to adjunctive risperidone in the Veterans Affairs Cooperative Study #504. J Clin Psychiatry. 2016;77(4):483–491. 26890894 10.4088/JCP.14m09585

[17] Lee DJ, Schnitzlein CW, Wolf JP, Vythilingam M, Rasmusson AM, Hoge CW. Psychotherapy versus pharmacotherapy for posttraumatic stress disorder: systemic review and meta-analyses to determine first-line treatments. Depress Anxiety. 2016;33(9):792–806. 27126398 10.1002/da.22511

[18] Watts BV, Schnurr PP, Mayo L, Young-Xu Y, Weeks WB, Friedman MJ. Meta-analysis of the efficacy of treatments for posttraumatic stress disorder. J Clin Psychiatry. 2013;74(6):e541–e550. 23842024 10.4088/JCP.12r08225

[19] Hamner MB, Hernandez-Tejada MA, Zuschlag ZD, Agbor-Tabi D, Huber M, Wang Z. Ziprasidone augmentation of SSRI antidepressants in posttraumatic stress disorder: a randomized, placebo-controlled pilot study of augmentation therapy. J Clin Psychopharmacol. 2019;39(2):153–157. doi:10.1097/JCP.0000000000001000 30640209

[20] Raskind MA, Peskind ER, Kanter ED, Reduction of nightmares and other PTSD symptoms in combat veterans by prazosin: a placebo-controlled study. Am J Psychiatry. 2003;160(2):371–373. 12562588 10.1176/appi.ajp.160.2.371

[21] Raskind MA, Peskind ER, Hoff DJ, A parallel group placebo controlled study of prazosin for trauma nightmares and sleep disturbance in combat Veterans with post-traumatic stress disorder. Biol Psychiatry. 2007;61(8):928–934. 17069768 10.1016/j.biopsych.2006.06.032

[22] Raskind MA, Peterson K, Williams T, A trial of prazosin for combat trauma PTSD with nightmares in active-duty soldiers returned from Iraq and Afghanistan. Am J Psychiatry. 2013;170(9):1003–1010. 23846759 10.1176/appi.ajp.2013.12081133

[23] Schneier FR, Campeas R, Carcamo J, Combined mirtazapine and SSRI treatment of PTSD: a placebo-controlled trial. Depress Anxiety. 2015;32(8):570–579. doi:10.1002/da.22384 26115513 PMC4515168

[24] Hadlandsmyth K, Bernardy NC, Lund BC. Central nervous system polytherapy among veterans with posttraumatic stress disorder: changes across a decade [Published online ahead of print, 2021 Dec 10]. Gen Hosp Psychiatry. 2021;74:46–50. doi:10.1016/j.genhosppsych.2021.12.002 34906798

[25] Hofmeyer BA, Look KA, Hager DR. Refill-based medication use quality measures in kidney transplant recipients: examination of proportion of days covered and medication possession ratio. J Manag Care Spec Pharm. 2018;24(4):367–372. doi:10.18553/jmcp.2018.24.4.367 29578851 PMC10398127

[26] Blanchard EB, Jones-Alexander J, Buckley TC, Forneris CA. Psychometric properties of the PTSD checklist (PCL). Behav Res Ther. 1996;34(8):669–673. 8870294 10.1016/0005-7967(96)00033-2

[27] Weathers FWL, Herman BT, Huska DS, Keane JA, TM. The PTSD checklist: reliability, validity, and diagnostic utility. Paper presented at: Annual Meeting for the International Society for Traumatic Stress Studies; 1993.

[28] McDonald SD, Calhoun PS. The diagnostic accuracy of the PTSD checklist: a critical review. Clin Psychol Rev. 2010;30(8):976–987. 20705376 10.1016/j.cpr.2010.06.012

[29] Greenberg G, Hoff R . Office of Mental Health Operations Mental Health Services Coding Rules. In. West Haven, CT: Northeast Program Evaluation Center. 2014.

[30] Tschoner A, Engl J, Laimer M, Metabolic side effects of antipsychotic medication. Int J Clin Pract. 2007;61(8):1356–1370. doi:10.1111/j.1742-1241.2007.01416.x 17627711

[31] Wang S, Linkletter C, Dore D, Mor V, Buka S, Maclure M. Age, antipsychotics, and the risk of ischemic stroke in the Veterans Health Administration. Stroke. 2012;43(1):28–31. doi:10.1161/STROKEAHA.111.617191 22033997

[32] Shiner B, Westgate CL, Gui J, A retrospective comparative effectiveness study of medications for posttraumatic stress disorder in routine practice. J Clin Psychiatry. 2018;79(5):18m12145. doi:10.4088/JCP.18m12145. PMC6217812 30257081

[33] Rothbaum BO, Davidson JR, Stein DJ, A pooled analysis of gender and trauma-type effects on responsiveness to treatment of PTSD with venlafaxine extended release or placebo. J Clin Psychiatry. 2008;69(10):1529–1539. doi:10.4088/jcp.v69n1002 19192435

[34] Germain A, Richardson R, Moul DE, Placebo-controlled comparison of prazosin and cognitive-behavioral treatments for sleep disturbances in US military veterans. J Psychosom Res. 2012;72(2):89–96. 22281448 10.1016/j.jpsychores.2011.11.010 PMC3267960

[35] Jonas DE, Cusack K, Forneris CA, Psychological and Pharmacological Treatments for Adults With Posttraumatic Stress Disorder (PTSD); Agency for Healthcare Research and Quality (US). 2013. 23658937

[36] Khachatryan D, Groll D, Booij L, Sepehry AA, Schutz CG. Prazosin for treating sleep disturbances in adults with posttraumatic stress disorder: a systematic review and meta-analysis of randomized controlled trials. Gen Hosp Psychiatry. 2016;39:46–52. 26644317 10.1016/j.genhosppsych.2015.10.007

[37] Raskind MA, Peskind ER, Chow B, Trial of prazosin for posttraumatic stress disorder in military veterans. N Engl J Med. 2018;378(6):507–517. doi:10.1056/NEJMoa1507598 29414272

[38] Serretti A, Mandelli L. Antidepressants and body weight: a comprehensive review and meta-analysis. J Clin Psychiatry. 2010;71(10):1259–1272. doi:10.4088/JCP.09r05346blu 21062615

[39] Rummel-Kluge C, Komossa K, Schwarz S, Head-to-head comparisons of metabolic side effects of second generation antipsychotics in the treatment of schizophrenia: a systematic review and meta-analysis. Schizophr Res. 2010;123(2-3):225–233. doi:10.1016/j.schres.2010.07.012 20692814 PMC2957510

[40] Hamer M, Batty GD, Seldenrijk A, Kivimaki M. Antidepressant medication use and future risk of cardiovascular disease: the Scottish Health Survey [published correction appears in eur heart J. 2013 Oct;34(40):3160. David Batty, G (corrected to Batty, G David)]. Eur Heart J. 2011;32(4):437–442. doi:10.1093/eurheartj/ehq438 21118851 PMC3038336

[41] Bullock J, Whiteley C, Moakes K, Clarke I, Riches S. Single-session Comprehend, Cope, and Connect intervention in acute and crisis psychology: a feasibility and acceptability study. Clin Psychol Psychother. 2021;28(1):219–225. doi:10.1002/cpp.2505 32833291

[42] Killaspy H, Bebbington P, Blizard R, The REACT study: randomised evaluation of assertive community treatment in north London. BMJ. 2006;332(7545):815–820. doi:10.1136/bmj.38773.518322.7C 16543298 PMC1432213

[43] Blain RC, Pukay-Martin ND, Martin CE, Dutton-Cox CE, Chard KM. Residential cognitive processing therapy decreases suicidality by reducing perceived burdensomeness in veterans with posttraumatic stress disorder. J Trauma Stress. 2021;34(6):1199–1208. doi:10.1002/jts.22618 33128808

[44] Holliday R, Holder N, Monteith LL, Surís A. Decreases in suicide cognitions after cognitive processing therapy among veterans with posttraumatic stress disorder due to military sexual trauma: a preliminary examination. J Nerv Ment Dis. 2018;206(7):575–578. doi:10.1097/NMD.0000000000000840 29905663

[45] Norr AM, Smolenski DJ, Reger GM. Effects of prolonged exposure and virtual reality exposure on suicidal ideation in active duty soldiers: an examination of potential mechanisms. J Psychiatr Res. 2018;103:69–74. doi:10.1016/j.jpsychires.2018.05.009 29783077

[46] de Bont PA, van den Berg DP, van der Vleugel BM, Prolonged exposure and EMDR for PTSD v. a PTSD waiting-list condition: effects on symptoms of psychosis, depression and social functioning in patients with chronic psychotic disorders. Psychol Med. 2016;46(11):2411–2421. doi:10.1017/S0033291716001094 27297048

