# Supplementary material for: Comparison of mental health outcomes of augmenting medications for patients with posttraumatic stress disorder: A national veterans affairs study
Source: J Eval Clin Pract. Author manuscript; Available in PMC 2026 Apr 8. (PMC13058844; doi:10.1111/jep.13726)
Supplement: Supplement [file NIHMS2157918-supplement-Supplement.docx]

**Supplementary Materials**

**Supplementary Table 1**

*Propensity Score Weighted Changes in Percent of Patients Hospitalized in Total Sample and By Augmenting Medication Group*

|  | Medication Group | Pre-Index Year | Post-Index Year | Change (post minus pre) | 95% CI for Absolute Change | % Change | P-value |
| --- | --- | --- | --- | --- | --- | --- | --- |
| Mental Health  ER Visits  (% of patients) |  |  |  |  |  |  |  |
|  | All | 6.08 | 4.32 | -1.77 | (1.59 ,1.95) | 29.04 | <.0001 |
|  | Antipsychotics | 7.83 | 5.45 | -2.38 | (1.99 ,2.77) | 30.42 | <.0001 |
|  | Mirtazapine | 6.43 | 4.63 | -1.80 | (1.4 ,2.2) | 28.00 | <.0001 |
|  | Prazosin | 5.80 | 3.99 | -1.81 | (1.55 ,2.07) | 31.19 | <.0001 |
|  | Tricyclics | 4.23 | 3.17 | -1.06 | (0.55 ,1.57) | 25.06 | <.0001 |
| Mental Health Hospitalizations (% of patients) |  |  |  |  |  |  |  |
|  | All | 7.75 | 5.11 | -2.64 | (2.44 ,2.84) | 34.07 | <.0001 |
|  | Antipsychotics | 10.31 | 6.89 | -3.42 | (2.99 ,3.86) | 33.18 | <.0001 |
|  | Mirtazapine | 7.86 | 5.28 | -2.58 | (2.14 ,3.02) | 32.79 | <.0001 |
|  | Prazosin | 7.58 | 4.90 | -2.67 | (2.38 ,2.97) | 35.30 | <.0001 |
|  | Tricyclics | 5.19 | 3.32 | -1.87 | (1.32 ,2.42) | 35.99 | <.0001 |

**Supplementary Table 2**

*Coding Algorithms*

| **Variable** | **IDC9 Codes** | **ICD10 Codes** |
| --- | --- | --- |
| PTSD | 309.81 | F43.1x |
| Bipolar Disorder | 296.0x, 296.1x, 296.4x, 296.5x, 296.6x, 296.7, 296.8x | F30.xx, F31.xx |
| Any Psychotic Disorder | 295.xx, 298.0, 298.1, 298.4, 298.8, 298.9, 293.81, 293.82, 296.24, 296.34 | F06.0, F06.2, F20.xx, F22, F23, F24, F25.x, F28, F29, F32.3, F33.3, F53 |
| Major Depressive Disorder | 296.2x, 296.3x | F32.0, F32.1, F32.2, F32.3, F32.4, F32.5, F32.9, F33.0, F33.1, F33.2, F33.3, F33.4x, F33.9 |
| Other Depressive | 293.83, 296.9x, 298.0, 300.4, 301.11, 301.12, 301.13, 309.0, 309.1, 311 | F06.31, F06.362, F32.89, F33.8, F34.1 |
| Substance Use Disorders | 291.xx, 292.xx, 303.xx, 304.xx, 305.0x, 305.2x, 305.3x, 305.4x, 305.5x, 305.6x, 305.7x, 305.8x, 305.9x | F10.10, F10.12x, F10.14, F10.15x, F10.18x, F10.19, F10.2xx, F10.9xx, F11.10, F11.12x, F11.14, F11.15x, F11.18x, F11.19, F11.2xx, F11.9xx, F12.10, F12.12x, F12.15x, F12.18x, F12.19, F12.20, F12.21, F12.22x, F12.25x, F12.28x, F12.29, F12.90, F12.92x, F12.95x, F12.98x, F12.99, F13.10, F13.12x, F13.14, F13.15x, F13.18x, F13.19, F13.2xx, F13.9xx, F14.10, F14.12x, F14.14, F14.15x, F14.18x, F14.19, F14.2xx, F14.9xx, F15.10, F15.12x, F15.14, F15.15x, F15.18x, F15.19, F15.2xx, F15.9xx, F16.10, F16.12x, F16.14, F16.15x, F16.18x, F16.19, F16.2xx, F16.9xx, F18.10, F18.12x, F18.14, F18.15x, F18.17, F18.18x, F18.19, F18.2xx, F18.9xx, F19.10, F19.12x, F19.14, F19.15x, F19.16, F19.17, F19.18x, F19.19, F19.2xx, F19.9xx |
| Alcohol Use Disorders | 291.xx, 303.xx, 305.0x | F10.10, F10.12x, F10.14, F10.15x, F10.18x, F10.19, F10.2xx, F10.9xx |
| Personality Disorders | 301.x | F21, F60.xx, F69 |
| Anxiety | 300.02 | F41.1 |
| Insomnia | 307.41, 307.42, 307.49, 327.01, 372.02, 327.09, 780.52 | F51.0x, G47.0x |
| Obesity | 278.00, 278.01, 278.02 | E66.01, E66.3, E66.9 |
| Dislipidemia | 272.0, 272.1, 272.2, 272.3, 272.4, 272.7, 272.8, 272.9 | E75.21, E75.22, E75.249, E77.0, E77.1, E78.0x, E78.1, E78.2, E78.3, E78.4, E78.5, E78.8x, E78.9 |
| Diabetes | 250.xx | E10.1x, E10.29, E10.31x, E10.36, E10.37Xx, E10.39, E10.40, E10.51, E10.618, E10.62x, E10.63x, E10.64x, E10.65, E10.69, E10.8, E10.9, E11.0x, E11.21, E11.29, E11.31x, E11.36, E11.39, E11.40, E11.51, E11.618, E11.62x, E11.62x, E11.64x |
| Hypertension | 401.x, 402.xx, 403.xx, 404.xx, 405.xx | I10, I11.x, I12.x, I13.xx, I15.0, I15.8, I16.9 |
| Heart Disease | 410.xx, 411.xx, 413.x | I20.1, I20.8, I20.9, I21.09, I21.1x, I21.2x, I21.3, I21.4, I24.0, I24.1, I24.8 |
| Heart Failure | 428.xx | I50.1, I50.2x, I50.3x, I50.4x, I50.9 |
| Cardiovascular Disease | 430, 431, 433.01, 433.11, 433.21,433.31, 433.81, 433.91, 434.01, 434.11, 434.91, 436 | I60.9, I61.9, I63.019, I63.119, I63.139, I63.20, I63.219, I63.22, I63.239, I63.30, I63.40, I63.50, I63.59, I67.89 |

**Supplementary Table 3**

*Patient Characteristics by Augmenting Medication Class: Unweighted*

|  | Antipsychotics | Mirtazapine | Prazosin | Tricyclics | P-value |
| --- | --- | --- | --- | --- | --- |
| Sociodemographics | | | | | |
| Age | 51.9/14.4 | 53.9/14.2 | 51.5/14.3 | 52.1/13.2 | <.001 |
| Male | 92.2 | 91.8 | 92.3 | 84.8 | <.001 |
| Female | 7.8 | 8.2 | 7.7 | 15.2 |  |
| Race (White) | 73.8 | 74.5 | 72.2 | 73.6 | <.001 |
| Race (Black) | 19.1 | 18.7 | 19.4 | 19.4 |  |
| Race (Other) | 7.1 | 6.9 | 8.4 | 7.0 |  |
| Ethnicity (Hispanic) | 7.3 | 6.3 | 8.3 | 6.1 | <.001 |
| Ethnicity (Non-Hispanic) | 90.7 | 91.7 | 89.5 | 92.1 |  |
| Ethnicity (Unknown) | 2.0 | 2.0 | 2.2 | 1.9 |  |
| Marital Status (Married) | 56.2 | 56.6 | 59.9 | 58.5 | <.001 |
| Marital Status (Never Married) | 13.5 | 11.9 | 12.1 | 11.7 |  |
| Marital Status (Other) | 30.3 | 31.4 | 28.0 | 29.8 |  |
| Comorbidities | | | | | |
| Major Depressive Disorder | 37.1 | 39.1 | 35.5 | 34.5 | <.001 |
| Personality Disorder | 6.7 | 4.8 | 4.0 | 4.4 | <.001 |
| Generalized Anxiety Disorder | 8.2 | 8.3 | 6.9 | 7.2 | <.001 |
| Insomnia | 17.2 | 18.9 | 18.1 | 19.3 | <.001 |
| Substance abuse/dependence | 36.6 | 34.9 | 32.4 | 26.2 | <.001 |
| Alcohol abuse/dependence | 29.8 | 28.6 | 27.4 | 20.4 | <.001 |
| Traumatic Brain Injury | 6.3 | 5.3 | 6.4 | 6.6 | <.001 |
| Obesity | 24.0 | 23.1 | 28.8 | 29.0 | <.001 |
| Dyslipidemia | 52.5 | 57.2 | 55.4 | 57.3 | <.001 |
| Diabetes | 21.1 | 23.8 | 23.4 | 28.5 | <.001 |
| Hypertension | 52.7 | 56.8 | 54.1 | 56.3 | <.001 |
| Ischemic Heart Disease | 3.7 | 4.3 | 3.0 | 4.0 | <.001 |
| Congestive Heart Failure | 3.4 | 4.3 | 2.5 | 3.5 | <.001 |
| Cerebrovascular disease | 1.1 | 1.5 | 1.0 | 1.3 | <.001 |
| Charlson Comorbidity Index score | 1.1/1.7 | 1.3/1.8 | 1/1.6 | 1/1.8 | <.001 |
| Service Utilization Factors | | | | | |
| Primary Facility (VA Medical Center) | 62.3 | 60.7 | 61.5 | 64.1 | <.001 |
| Primary Facility (Community-Based Outpatient Clinic) | 37.7 | 39.3 | 38.5 | 35.9 |  |
| Primary care utilization  (# visits in pre-index year) | 3.8/3.8 | 3.9/3.8 | 3.6/3.5 | 4.4/4.1 | <.001 |
| Mental health utilization  (# visits in pre-index year) | 17.8/31.6 | 16.8/30.5 | 16.5/28.5 | 13.7/26.1 | <.001 |
| Drive time to nearest VA | 23.3/24.2 | 23.3/20.4 | 23.4/29.8 | 24.1/31.2 | <.001 |
| VA Service Connection level | 51.5/36.5 | 51.1/36.3 | 53.4/34.8 | 55/35.6 | <.001 |

*Note.* Values are shown as percent for categorical variables and mean/standard deviation for continuous variables.

**Supplementary Table 4**

*Unadjusted Models for Change in Outcomes by Augmenting Medication Group*

| **Variable** | **Medication** | **Diff to Ref** | **95% CI** | **P-value** |
| --- | --- | --- | --- | --- |
| PTSD Checklist |  |  |  |  |
|  | Mirtazapine | 0.02 | (-0.36,0.40) | 0.92 |
|  | Prazosin | -0.32 | (-0.63,-0.01) | 0.04 |
|  | Tricyclics | 0.02 | (-0.53,0.57) | 0.95 |
| Mental Health ER |  |  |  |  |
|  | Mirtazapine | 0.71 | (-11.15, 12.57) | 0.91 |
|  | Prazosin | 1.04 | (-4.67, 6.75) | 0.72 |
|  | Tricyclics | 2.77 | (-0.22, 5.77) | 0.07 |
| Mental Health Hospitalizations |  |  |  |  |
|  | Mirtazapine | 1.86 | (-0.8, 4.52) | 0.17 |
|  | Prazosin | 1.64 | (-5.13, 8.40) | 0.63 |
|  | Tricyclics | 4.09 | (0.23, 7.95) | 0.04 |

*Note*. Model shows coefficient for difference in change in outcome score from pre- to post index year as compared to reference group of Antipsychotics.

**Supplementary Table 5**

*PCL Checklist Age and Sex Subgroup Analyses with Antipsychotic Group as Reference, Propensity Weighted Models*

| **Subgroups** | **Medication Group** | **Diff to Ref** | **Three Way Interaction** | **P-value for 3-way Interaction** |
| --- | --- | --- | --- | --- |
| Sex |  |  |  |  |
|  | Mirtazapine: male | 0.004 |  |  |
|  | Mirtazapine: female | -0.01 |  |  |
|  |  |  | Time X Sex X Medication Group (Mirtazapine vs. Antipsychotic) | 0.98 |
|  | Prazosin: male | -0.36 |  |  |
|  | Prazosin: female | 0.11 |  |  |
|  |  |  | Time X Sex X Medication Group (Prazosin vs. Antipsychotic) | 0.44 |
|  | Tricyclics: male | -0.18 |  |  |
|  | Tricyclics: female | 0.23 |  |  |
|  |  |  | Time X Sex X Medication Group (Tricyclics vs. Antipsychotic) | 0.54 |
| Age |  |  |  |  |
|  | Mirtazapine: 64 and younger | -0.01 |  |  |
|  | Mirtazapine: 65 and older | 0.03 |  |  |
|  |  |  | Time X Age X Medication Group (Mirtazapine vs. Antipsychotic) | 0.95 |
|  | Prazosin: 64 and younger | -0.30 |  |  |
|  | Prazosin: 65 and older | -0.61 |  |  |
|  |  |  | Time X Age X Medication Group (Prazosin vs. Antipsychotic) | 0.68 |
|  | Tricyclics: 64 and younger | -0.26 |  |  |
|  | Tricyclics: 65 and older | 1.63 |  |  |
|  |  |  | Time X Age X Medication Group (Tricyclics vs. Antipsychotic) | 0.03 |

*Note*. Model shows coefficient for difference in change in outcome score from pre- to post index year as compared to the corresponding reference subgroup of Antipsychotics.

**Supplementary Table 6**

*Mental Health ER Visits (# per 100 person years)* *Age and Sex Subgroup Analyses with Antipsychotic Group as Reference, Propensity Weighted Models*

| **Subgroups** | **Medication Group** | **Diff to Ref** | **Three Way Interaction** | **P-value for 3-way Interaction** |
| --- | --- | --- | --- | --- |
| Sex |  |  |  |  |
|  | Mirtazapine: male | 0.01 |  |  |
|  | Mirtazapine: female | -0.02 |  |  |
|  |  |  | Time X Sex X Medication Group (Mirtazapine vs. Antipsychotic) | 0.15 |
|  | Prazosin: male | 0.01 |  |  |
|  | Prazosin: female | -0.01 |  |  |
|  |  |  | Time X Sex X Medication Group (Prazosin vs. Antipsychotic) | 0.40 |
|  | Tricyclics: male | 0.02 |  |  |
|  | Tricyclics: female | 0.01 |  |  |
|  |  |  | Time X Sex X Medication Group (Tricyclics vs. Antipsychotic) | 0.56 |
| Age |  |  |  |  |
|  | Mirtazapine: 64 and younger | 0.002 |  |  |
|  | Mirtazapine: 65 and older | 0.01 |  |  |
|  |  |  | Time X Age X Medication Group (Mirtazapine vs. Antipsychotic) | 0.65 |
|  | Prazosin: 64 and younger | 0.002 |  |  |
|  | Prazosin: 65 and older | 0.01 |  |  |
|  |  |  | Time X Age X Medication Group (Prazosin vs. Antipsychotic) | 0.65 |
|  | Tricyclics: 64 and younger | 0.01 |  |  |
|  | Tricyclics: 65 and older | 0.04 |  |  |
|  |  |  | Time X Age X Medication Group (Tricyclics vs. Antipsychotic) | <.001 |

*Note*. Model shows coefficient for difference in change in outcome score from pre- to post index year as compared to the corresponding reference subgroup of Antipsychotics.

**Supplementary Table 7**

*Mental Health Hospitalizations (# per 100 person years) Age and Sex Subgroup Analyses with Antipsychotic Group as Reference, Propensity Weighted Models*

| **Subgroups** | **Medication Group** | **Diff to Ref** | **Three Way Interaction** | **P-value for 3-way Interaction** |
| --- | --- | --- | --- | --- |
| Sex |  |  |  |  |
|  | Mirtazapine: male | 0.01 |  |  |
|  | Mirtazapine: female | <0.001 |  |  |
|  |  |  | Time X Sex X Medication Group (Mirtazapine vs. Antipsychotic) | 0.41 |
|  | Prazosin: male | 0.01 |  |  |
|  | Prazosin: female | <0.001 |  |  |
|  |  |  | Time X Sex X Medication Group (Prazosin vs. Antipsychotic) | 0.86 |
|  | Tricyclics: male | 0.02 |  |  |
|  | Tricyclics: female | 0.02 |  |  |
|  |  |  | Time X Sex X Medication Group (Tricyclics vs. Antipsychotic) | 0.53 |
| Age |  |  |  |  |
|  | Mirtazapine: 64 and younger | 0.01 |  |  |
|  | Mirtazapine: 65 and older | 0.01 |  |  |
|  |  |  | Time X Age X Medication Group (Mirtazapine vs. Antipsychotic) | 0.58 |
|  | Prazosin: 64 and younger | 0.003 |  |  |
|  | Prazosin: 65 and older | 0.01 |  |  |
|  |  |  | Time X Age X Medication Group (Prazosin vs. Antipsychotic) | 0.70 |
|  | Tricyclics: 64 and younger | 0.01 |  |  |
|  | Tricyclics: 65 and older | 0.04 |  |  |
|  |  |  | Time X Age X Medication Group (Tricyclics vs. Antipsychotic) | <.001 |

*Note*. Model shows coefficient for difference in change in outcome score from pre- to post index year as compared to the corresponding reference subgroup of Antipsychotics.

**Supplementary Table 8**

*Models for Change in Outcomes by Augmenting Medication Group, Propensity Weighted and Adjusted for Time on Augmenting Medications, Dosage, and Exposure to Other Classes of Augmenting Medications*

| **Variable** | **Medication** | **Diff to Ref** | **95% CI** | **P-value** |
| --- | --- | --- | --- | --- |
| PTSD Checklist |  |  |  |  |
|  | Mirtazapine | -0.05 | (-0.42,0.31) | 0.77 |
|  | Prazosin | -0.38 | (-0.73,-0.03) | 0.03 |
|  | Tricyclics | -0.20 | (-0.58,0.17) | 0.29 |
| Mental Health ER |  |  |  |  |
|  | Mirtazapine | 1.35 | (-25.94, 28.64) | 0.92 |
|  | Prazosin | 0.53 | (-0.98, 2.05) | 0.49 |
|  | Tricyclics | 1.96 | (-1.41, 5.33) | 0.25 |
| Mental Health Hospitalizations |  |  |  |  |
|  | Mirtazapine | 2.63 | (-1.35, 6.62) | 0.20 |
|  | Prazosin | 0.68 | (-0.51, 1.87) | 0.26 |
|  | Tricyclics | 2.39 | (-6.67, 11.45) | 0.61 |

*Note*. Model shows coefficient for difference in change in outcome score from pre- to post index year as compared to reference group of Antipsychotics.

**Supplementary Table 9**

*Models for Change in Outcomes by Augmenting Medication Group, Propensity Weighted in Restricted Sample Using Only a Single Class of Augmenting Medication*

| **Variable** | **Medication** | **Diff to Ref** | **95% CI** | **P-value** |
| --- | --- | --- | --- | --- |
| PTSD Checklist |  |  |  |  |
|  | Mirtazapine | -0.34 | (-0.96, 0.29) | 0.29 |
|  | Prazosin | -0.68 | (-1.26, -0.09) | 0.02 |
|  | Tricyclics | -0.36 | (-1.02, 0.31) | 0.29 |
| Mental Health ER |  |  |  |  |
|  | Mirtazapine | 0.34 | (-3.01, 3.69) | 0.84 |
|  | Prazosin | 0.51 | (-1.42, 2.43) | 0.61 |
|  | Tricyclics | 1.20 | (-38.67, 41.07) | 0.95 |
| Mental Health Hospitalizations |  |  |  |  |
|  | Mirtazapine | 0.89 | (-81.46, 83.24) | 0.98 |
|  | Prazosin | 0.84 | (-1.87, 3.54) | 0.54 |
|  | Tricyclics | 1.16 | (0.31, 2.01) | 0.01 |

*Note*. Model shows coefficient for difference in change in outcome score from pre- to post index year as compared to reference group of Antipsychotics.

**Supplementary Figure 1**

*Overview of Study Design*

**Index Date**

Augmenting medication added

**Post-Index Year:** PTSD Symptoms, Mental Health Emergency Room Visits/Hospitalizations

**Pre-Index Year:** PTSD Symptoms, Mental Health Emergency Room Visits/Hospitalizations
